# Role for Tetrahydrobiopterin in the Fetoplacental Endothelial Dysfunction in Maternal Supraphysiological Hypercholesterolemia

**DOI:** 10.1155/2016/5346327

**Published:** 2015-11-30

**Authors:** Andrea Leiva, Bárbara Fuenzalida, Francisco Westermeier, Fernando Toledo, Carlos Salomón, Jaime Gutiérrez, Carlos Sanhueza, Fabián Pardo, Luis Sobrevia

**Affiliations:** ^1^Cellular and Molecular Physiology Laboratory (CMPL), Division of Obstetrics and Gynaecology, School of Medicine, Faculty of Medicine, Pontificia Universidad Católica de Chile, 8330024 Santiago, Chile; ^2^Advanced Center for Chronic Diseases (ACCDiS), Faculty of Chemical & Pharmaceutical Sciences and Faculty of Medicine, Universidad de Chile, 8380492 Santiago, Chile; ^3^Faculty of Science, Universidad San Sebastián, 7510157 Santiago, Chile; ^4^Department of Basic Sciences, Faculty of Sciences, Universidad del Bío-Bío, 3780000 Chillán, Chile; ^5^University of Queensland Centre for Clinical Research (UQCCR), Faculty of Medicine and Biomedical Sciences, University of Queensland, Herston, QLD 4029, Australia; ^6^Cellular Signaling and Differentiation Laboratory (CSDL), Health Sciences Faculty, Universidad San Sebastian, 7510157 Santiago, Chile; ^7^Department of Physiology, Faculty of Pharmacy, Universidad de Sevilla, 41012 Seville, Spain

## Abstract

Maternal physiological hypercholesterolemia occurs during pregnancy, ensuring normal fetal development. In some cases, the maternal plasma cholesterol level increases to above this physiological range, leading to maternal supraphysiological hypercholesterolemia (MSPH). This condition results in endothelial dysfunction and atherosclerosis in the fetal and placental vasculature. The fetal and placental endothelial dysfunction is related to alterations in the L-arginine/nitric oxide (NO) pathway and the arginase/urea pathway and results in reduced NO production. The level of tetrahydrobiopterin (BH_4_), a cofactor for endothelial NO synthase (eNOS), is reduced in nonpregnant women who have hypercholesterolemia, which favors the generation of the superoxide anion rather than NO (from eNOS), causing endothelial dysfunction. However, it is unknown whether MSPH is associated with changes in the level or metabolism of BH_4_; as a result, eNOS function is not well understood. This review summarizes the available information on the potential link between MSPH and BH_4_ in causing human fetoplacental vascular endothelial dysfunction, which may be crucial for understanding the deleterious effects of MSPH on fetal growth and development.

## 1. Introduction

Hypercholesterolemia is considered one of the most important risk factors for the development of cardiovascular disease (Adult Treatment Panel ATP III) [1, 2]. Pregnancy is a physiological process that can involve the development of maternal pathologies, such as preeclampsia, gestational diabetes mellitus (GDM), and metabolic disorders, including maternal pregestational obesity, supraphysiological gestational weight gain (spGWG) [3, 4], and hypercholesterolemia [5, 6]. These alterations may compromise the health of the mother and/or the fetus [5–7]. In normal pregnancies, the mother exhibits a physiological (i.e., normal) increase (30–50%) in the plasma total cholesterol (TCh) level in a process that is referred to as maternal physiological hypercholesterolemia (MPH) [5, 6, 8]. However, in some cases, for mostly unknown reasons, the TCh level is elevated far beyond the physiological range, which is referred to as maternal supraphysiological hypercholesterolemia (MSPH) [5, 6, 9]. Although studies have shown the potential adverse effects of MSPH on the early development of atherosclerosis [10, 11] and endothelial dysfunction in the fetoplacental vasculature [5, 6], the global prevalence of MSPH remains unknown [12].

Endothelial cells synthetize nitric oxide (NO), a potent vasodilator that is generated by NO synthases (NOS; i.e., the L-arginine/NO pathway), following the oxidation of L-arginine in a process that depends on the bioactivity of several cofactors, including tetrahydrobiopterin (BH_4_) [13–15]. In pregnant women with MSPH, the umbilical vein dilation and endothelial NOS (eNOS) activity are reduced, and arginase (ARG) activity is increased compared with MPH [5, 6]. Remarkably, ARG inhibition results in a partial restoration of human umbilical vein dilation, suggesting that other factors play a role in this phenomenon. In nonpregnant women, hypercholesterolemia reduces the NO bioavailability by mechanisms that include a reduction in BH_4_ levels [16]. Reduced activity and/or level of BH_4_ favor(s) the generation of a superoxide anion (O_2_^∙−^) instead of NO, resulting in endothelial dysfunction [14, 17, 18]. Altogether, this indicates that elevated plasma TCh levels may result in reduced NO synthesis via several mechanisms, leading to endothelial dysfunction. The potential effect of MSPH on BH_4_ availability and regulation of the biosynthesis of this cofactor, as well as its consequences in the modulation of fetal endothelial function, are unknown [5, 6]. This review summarizes the findings regarding a potential link between MSPH and BH_4_ and human fetoplacental vascular endothelial dysfunction.

## 2. Hypercholesterolemia

In nonpregnant women, hypercholesterolemia is mainly related to genetic mutations involving genes that are related to cholesterol traffic, such as lipoprotein receptors and cholesterol transporters, metabolic disorders, and an imbalanced diet [2, 19]. According to the Third Report of the National Cholesterol Education Program (NCEP) Expert Panel on Detection, Evaluation, and Treatment of High Blood Cholesterol in Adults (Adult Treatment Panel III), this condition is the main risk factor for the development of cardiovascular disease (CVD) [1, 2]. Therefore, the proper management of plasma cholesterol levels can reduce the risk of developing CVD [20]. For this reason, the clinical* cut-off* point for normal blood TCh in nonpregnant women is rigorously controlled and suggested to be <200 mg/dL [1].

A higher risk of hypercholesterolemia-associated CVD also results from a reduced blood level of high-density lipoprotein cholesterol (HDL, i.e., <40 mg/dL) and/or an increased blood level of low-density lipoprotein cholesterol (LDL, i.e., >100 mg/dL); the latter depends on the global cardiovascular risk, as recently recommended in the Guideline on the Treatment of Blood Cholesterol to Reduce Atherosclerotic Cardiovascular Risk in Adults from the American Heart Association [21]. Although CVD is normally diagnosed in adults, some studies indicate that endothelial dysfunction (i.e., an initial phenomenon in the development of atherosclerosis) and early atherosclerotic lesions (i.e., fatty streaks) begin during intrauterine life, in fetal vessels, as a consequence of increased levels of maternal cholesterol [5, 6, 10]. This indicates the relevance of monitoring the potential adverse effects of maternal cholesterolemia during pregnancy on the fetal vasculature [22]. This is even more important because the current global prevalence for a high plasma concentration of TCh (>200 mg/dL) in nonpregnant women is ~40% [23]. It is conceivable that a significant number of pregnant women will have increased plasma levels of cholesterol, exposing them to the inherent consequences of this condition as well as the associated fetal vascular alterations [12].

*Hypercholesterolemia during Pregnancy*. Pregnancy is a physiological condition that is characterized by progressive weeks of gestation-dependent increases (increasing 40–50%) in the maternal blood level of cholesterol and triglycerides [24, 25]. MPH is considered an adaptive response of the mother to satisfy the high cholesterol demand of the growing fetus for membrane and hormone synthesis [26]. Unfortunately, there are no established clinical reference values for the total and lipoprotein cholesterol levels during pregnancy in the global population, including the pregnant Chilean population [5, 6, 8]. A summary of the literature on the TCh, lipoprotein cholesterol, and triglyceride concentrations per trimester of pregnancy for different populations is shown in Table 1. Based on the literature, the estimated mean values for TCh are 179, 226, and 257 mg/dL for the 1st (1–13 weeks of pregnancy), 2nd (14–28 weeks of pregnancy), and 3rd (28–40 weeks of pregnancy) trimesters of pregnancy, respectively. For HDL and LDL, the mean values were 62 and 101, 73 and 131, and 68 and 149 mg/dL for the 1st, 2nd, and 3rd trimesters of pregnancy, respectively. MSPH has been defined by considering an at-term* cut-off* point for TCh of 280–300 mg/dL [5, 11, 27], and it is associated with vascular alterations at birth [5] and during childhood [28]. Additionally, increased oxidative stress in the maternal and fetal blood and placenta [27] as well as reduced expression of placental LDL receptors [29] was found in pregnancies that had maternal TCh levels that were higher than this* cut-off* point.

Although lipid trafficking through the placenta is restrictive and children born to mothers with MSPH have normal blood cholesterol levels [5, 31], a positive correlation between the maternal and fetal blood cholesterol in the 2nd and 3rd trimesters of pregnancy has been established [11, 32]. Moreover, increased early atherosclerotic markers, such as fatty streaks and lipid peroxidation, were found in the aortas of human fetuses [11] as well as in 7- to 14-year-old children [12] who were born to mothers with MSPH. Furthermore, endothelial dysfunction in the human umbilical vein from pregnancies with TCh values that were higher than this* cut-off* point has been proposed to be associated with reduced endothelium dependent vascular relaxation, due to lower eNOS and higher ARG activity [5]. These results provide evidence for the potential effect of MSPH in the placenta, leading to adverse consequences for the fetal vasculature. Interestingly, there is limited information on the prevalence of MSPH in the global population, which is mainly because the maternal blood cholesterol level is not routinely evaluated during pregnancy. Moreover, in a group of pregnant Chilean women, the prevalence of this maternal condition was ~30% [5, 6]. As a result, a significantly higher number of pregnant women will potentially present with an adverse intrauterine condition that could result in the development of vascular alterations in the growing fetus, such as endothelial dysfunction and early atherosclerosis.

## 3. Fetoplacental Endothelial Dysfunction in MSPH

The placenta is a physical and metabolic barrier between the fetal and maternal circulations, and it is a crucial organ that supports proper fetal development [33]. Because the placenta and umbilical cord lack autonomic innervation [34], a balance between circulating vasodilators and vasoconstrictors is crucial to maintaining normal fetoplacental function [33, 35]. Endothelial dysfunction is defined as an imbalance between vasodilator and vasoconstrictor molecules that are produced by or acting on endothelial cells [36] and that are critical for normal fetal development.

### 3.1. L-Arginine/NO Pathway

NO is a gas derived from the metabolism of L-arginine via the enzyme NOS, in a metabolic reaction where there is equimolar generation of L-citrulline and NO (as in the L-arginine/NO pathway) [7]. NOS are a group of enzymes with at least three isoforms that are encoded by different genes in mammals [57], that is, neuronal NOS (nNOS or type 1), inducible NOS (iNOS or type 2), and endothelial NOS (eNOS or type 3). eNOS is the main form that is expressed in endothelial cells [3], and reduced activity of this enzyme may result from lower expression, reduced activation, or increased inactivation [58, 59]. eNOS activity is modulated by different agents, including the level of its cofactor BH_4_ and posttranslational phosphorylation/dephosphorylation. For example, phosphorylation of serine 1177 (Ser^1177^) via PI3 kinase/Akt is associated with higher activity [60]; however, phosphorylation of threonine 495 (Thr^495^) via protein kinase C (PKC) maintains a low activity of this enzyme [58, 61]. Remarkably, hypercholesterolemia is associated with reduced eNOS expression, an effect that is reversed by restoring the cholesterol levels with the use of statins, for example, [62–64]. Additionally, it has been shown that cholesterol regulates the phosphorylation of eNOS. In mice and pigs, there is a negative correlation between the TCh level and the activation-phosphorylation of Ser^1177^ [65, 66]. On the other hand, HDL also induces Ser^1177^ phosphorylation of eNOS [67]. It was recently shown that, in fetoplacental macrovascular endothelial cells from pregnant women with MSPH, eNOS activity, but not its protein abundance, is reduced [5]. However, Ser^1177^and Thr^495^phosphorylation was reduced compared to cells from pregnant women with MPH. As a result, an altered maternal cholesterol level may modify the eNOS activity in pregnancy.

### 3.2. ARGs/Urea Pathway

ARGs (ARG-I and ARG-II) are a family of enzymes that compete with NOS for the substrate L-arginine, leading to the synthesis of L-ornithine and urea [68]. Interestingly, hypercholesterolemia is associated with increased ARG activity in animal models [69, 70] and humans [71, 72]. The activity of ARGs is also increased in HUVECs from pregnancies with MSPH compared with MPH pregnancies [5]. Because the pharmacological blockade of ARG with* S*-(2-boronoetil)-L-cysteine (BEC) partially reverses the reduced eNOS activity observed in HUVECs in MSPH pregnancies, ARGs are likely involved in modulating eNOS activity in this cell type [4].

## 4. BH_**4**_ Metabolism in MSPH

### 4.1. BH_4_

**Figure 1:**
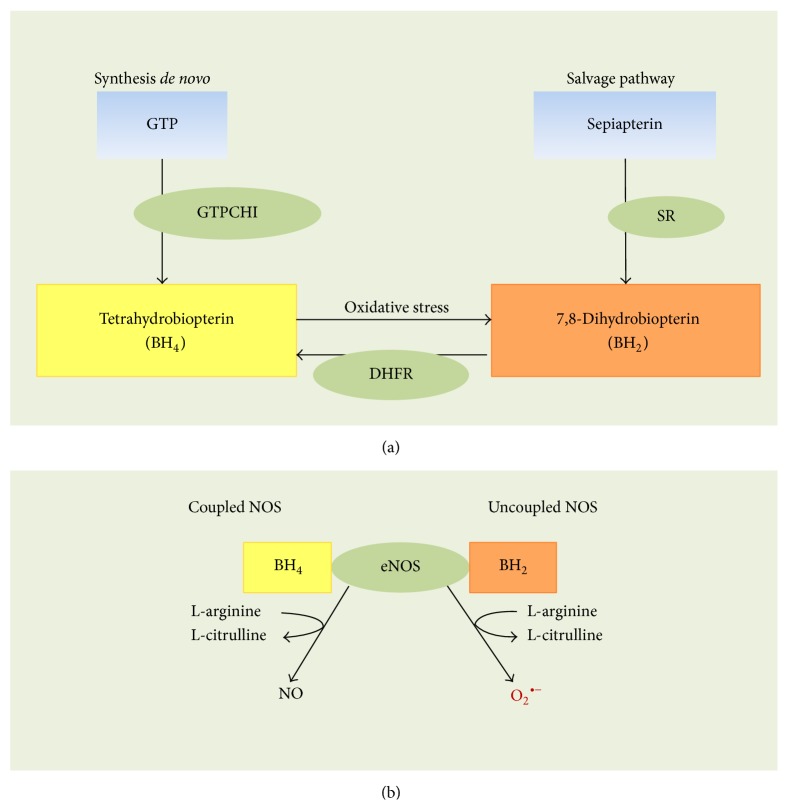
Tetrahydrobiopterin metabolism and endothelial nitric oxide synthase uncoupling. (a) The first step in the* de novo* synthesis of tetrahydrobiopterin (BH_4_) is the rate limiting reaction involving the enzyme GTP cyclohydrolase 1 (GTPCH1), whose substrate is GTP. An alternative* salvage pathway* for BH_4_ synthesis is the reduction of 7,8-dihydrobiopterin (BH_2_) to BH_4 _by the enzyme dihydrofolate reductase (DHFR). BH_2_ is generated from sepiapterin by the sepiapterin reductase enzyme (SR).* Oxidative stress* may be an environmental condition that promotes the oxidation of BH_4_ to BH_2_, decreasing the bioavailability of BH_4_. (b) Under physiological conditions, nitric oxide synthases (NOS,* coupled NOS*) generate nitric oxide, following the metabolism of L-arginine into L-citrulline in the presence of BH_4_. However, uncoupling NOS (*uncoupled eNOS*) with these enzymes may result in the generation of a superoxide anion (O_2_^∙−^). This phenomenon results from a deficiency in BH_4_ and an increased BH_2_ bioavailability (from data in [6, 17, 30]).

BH_4 _is a cofactor required for NOS activity because this molecule stabilizes the enzyme as an active dimer, allowing for optimal oxidation of L-arginine into NO [13, 15]. A reduction in the BH_4 _level leads to reduced eNOS activation, which is likely due to the uncoupling that results in the generation of superoxide anion (O_2_^∙−^) rather than NO, promoting vascular oxidative stress and endothelial dysfunction [14]. In the endothelium, BH_4_ is synthesized by at least two metabolic pathways:* de novo* biosynthesis from guanosine triphosphate (GTP) and the salvage pathway from sepiapterin to BH_2_ and BH_4_ [14] (Figure 1(a)).* De novo* biosynthesis involves the sequential action of GTP cyclohydrolase 1 (GTPCH1), 6-piruvoil tetrahydropterin synthase (PTPS), and sepiapterin reductase (SR). The GTPCH1 step is the limiting step of the pathway, and it is highly regulated at the transcriptional, translational, and posttranslational levels [73]. For the salvage pathway, the reduction of BH_2_ to BH_4_ is the limiting step and requires the enzyme dihydrofolate reductase (DHFR) [73]. The BH_4_ level could be reduced by decreased synthesis and by the oxidation of BH_4_ to BH_2_ via oxygen-derived reactive species and peroxynitrite (ONOO^−^), resulting in eNOS uncoupling (Figure 1(b)) [13, 18, 74]. The latter is a phenomenon that occurs in a variety of clinical conditions that are associated with vascular disease, including diabetes mellitus, hypertension, atherosclerosis [75–77], and hyperglycemia [18].

### 4.2. BH_4_ Metabolism in Hypercholesterolemia

Patients with hypercholesterolemia have low NO availability [78, 79] as well as a lower BH_4_ level (Table 2). Interestingly, oral or local supplementation with BH_4_ restores the impaired NO-dependent vasodilation in subjects with hypercholesterolemia [13, 52, 55, 80]. The association between human hypercholesterolemia and a reduced level of GTPCH1 has not yet been addressed [14, 18]. However, reduced eNOS activity is reversed by supplementation with the BH_4_ substrate sepiapterin or by GTPCH1 overexpression in mice [81–83]. As a result, this enzyme likely plays a role in hypercholesterolemia. Incubation with human LDL reduces NOS and GTPCH1 expression in rat vascular smooth muscle cells [84, 85]. Additionally, a reduced level of GTPCH1 due to hyperglycemia in human aortic endothelial cells decreases the BH_4_ level and NO synthesis, which is reversed by GTPCH1 overexpression [86]. Interestingly, and in corroboration with these findings, there are results showing similar changes in HUVECs that were subjected to the pharmacological induction of GTPCH1 expression [87].

The potential effect of MSPH on BH_4_ availability in the regulation of the synthesis of this cofactor and its effect on the modulation of fetal endothelial function are unknown [5, 6]. Because NO synthesis is reduced in the fetal endothelium from pregnancies with MSPH via mechanisms involving increased ARG but reduced eNOS activity, it is hypothesized that this maternal condition could also result in reduced BH_4 _bioavailability for NO synthesis. As a result, these potential mechanisms could explain the endothelial dysfunction and reduced vascular relaxation observed in MSPH. Preliminary results show that the BH_4_ level is reduced in HUVECs from MSPH [88] (Leiva A., Sobrevia L.,* unpublished*). However, it is unknown whether BH_4_ metabolism is altered in human fetoplacental endothelial cells in pregnancies with MSPH or whether restoration of the BH_4_ level improves the endothelial dysfunction in MSPH [4–6, 33].

## 5. Concluding Remarks

**Figure 2:**
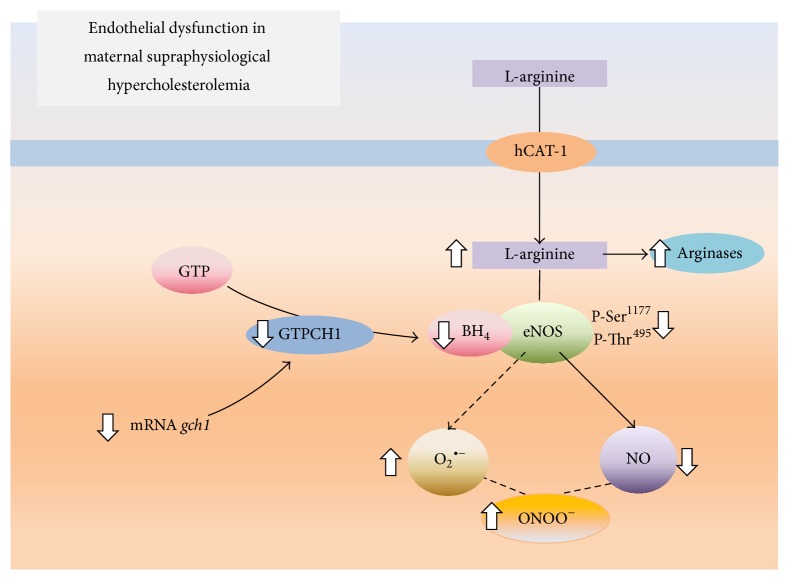
Effect of maternal supraphysiological hypercholesterolemia on the endothelial L-arginine/NO signaling pathway. In umbilical vein endothelial cells from pregnancies complicated by maternal physiological hypercholesterolemia, the amino acid L-arginine is taken up by the human cationic amino acid transporter 1 (hCAT-1) and metabolized by endothelial nitric oxide synthase (eNOS) and, to a lesser extent, arginases. This phenomenon occurs in the presence of tetrahydrobiopterin (BH_4_), resulting in NO generation. BH_4_ is generated by the enzyme GTP cyclohydrolase 1 (GTPCH1), which is coded by the* gch1* gene and whose substrate is GTP. In cells from pregnancies where the pregnant women had maternal supraphysiological hypercholesterolemia, hCAT-1-mediated L-arginine transport is increased (⇑), increasing the availability of this amino acid for eNOS and arginases. In this pathological condition, L-arginine is mainly used by arginases, limiting the formation of NO via eNOS. In addition, eNOS has reduced (⇓) activity because of the lower phosphorylation of Ser^1177^ and the bioavailability of BH_4_. The reduction in the BH_4_ concentration results from a reduced expression of* gch1*, leading to eNOS uncoupling and the generation of a superoxide anion (O_2_^∙−^). The O_2_^∙−^ reacts with NO to form peroxynitrite (ONOO^−^; from data in [5–7, 14]).

The prevalence of MSPH in the global population has not been evaluated, although it is estimated as approximately 30% [5, 6]. MSPH is a factor that favors the development of vascular changes in the growing fetus and, eventually, in children [12]. These vascular disorders include endothelial dysfunction in the fetus and placenta, disrupting the equilibration between the ARG/urea and L-arginine/NO signaling pathways. However, it is unknown whether these alterations correlate with the degree of MSPH in pregnancy or the alterations in BH_4_ metabolism and eNOS function (Figure 2). Drugs that control the TCh plasma level in adult, nonpregnant subjects are not used during pregnancy. This condition limits our present knowledge regarding the correlation between the mother's and fetus's TCh level and the vascular function of the fetus during pregnancy. However, based on the available evidence from subjects with hypercholesterolemia, we propose that restoration of the BH_4_ level will improve the fetoplacental endothelial function in humans. Therefore, it is essential to focus future studies on exploring the dynamics of the BH_4_ metabolism in MSPH pregnancies and the possible contribution that restoring this cofactor could have on this maternal condition and vascular function.

## Figures and Tables

**Table 1:** Reported maternal plasma lipid concentration in pregnancy.

Studied population (number of pregnant women)	Trimester of pregnancy	TCh	HDL	LDL	Tg	Observations	Reference
USA (142)	1 2 3	180 230 260	70 80 76	110 137 162	112 162 212	Maternal overweight and obesity association with lipid concentration during pregnancy	[37]

Brazil (288)	1 2 3	186 228 243	54 62 62	108 143 135	97 150 177	Maternal lipid concentration during pregnancy as a risk factor for GDM	[38]

Argentina (101)	1 2 3	160 201 244	58 62 61	78 107 144	90 140 202	Measurement of maternal plasma lipids during pregnancy	[39]

Chile (265)	1 2 3	178 232 269	60 73 75	102 124 147	108 179 244	Maternal lipid concentration association with impaired endothelium dependent dilation of the human umbilical vein	[6]

Chile (74)	1 2 3	— — 238	— — 72	— — 120	— — 232	Cut-off point for TCh in maternal plasma from where fetoplacental vascular dysfunction is seen	[5]

UK (178)	1 2 3	215 252 281	67 81 69	124 126 159	125 180 252	Measurement of maternal plasma lipids and apolipoproteins during pregnancy	[40]

UK (17)	1 2 3	164 212 261	— — —	— — —	77 133 233	Measurement of maternal plasma lipids and markers of oxidative stress in normal and GDM pregnancies	[41]

Ireland (222)	1 2 3	197 224 278	65 75 68	104 128 147	— — —	Reference values for maternal lipids during pregnancy	[42]

Italy (22)	1 2 3	178 247 282	68 73 68	97 153 168	93 155 230	Measurement of maternal plasma lipids during pregnancy	[43]

Sweden (18)	1 2 3	182 238 248	69 79 69	104 140 153	99 165 215	Measurement of maternal plasma lipids during pregnancy	[44]

Spain (45)	1 2 3	166 193 228	— — —	— — —	71 106 150	Measurement of maternal plasma LDL oxidation in normal, GDM, and obese pregnancies	[45]

Spain (25)	1 2 3	170 234 254	68 82 71	89 136 153	60 117 184	Maternal lipases activity and hormones concentrations during pregnancy	[46]

Serbia (50)	1 2 3	190 245 267	75 89 79	97 126 144	85 151 219	Maternal lipid concentration association with newborn size	[47]

Turkey (801)	1 2 3	166 — 271	53 — 63	94 — 155	93 — 274	Maternal lipid concentrations with fetal growth and development in GDM and preeclampsia	[48]

Israel (3938)	1 2 3	175 225 238	58 63 63	88 121 137	100 175 237	Association of maternal lipid concentration with preeclampsia and GDM	[49]

Nigeria (60)	1 2 3	172 203 232	41 51 63	112 126 136	93 128 171	Atherosclerotic risk in pregnant women	[50]

Women were subjected to lipids determination at 1st trimester (0–14 weeks of gestation), 2nd trimester (14–28 weeks of gestation), or 3rd trimester (28–40 weeks of gestation) of pregnancy. TCh: total cholesterol; LDL: low-density lipoprotein; HDL: high-density lipoprotein; Tg: triglycerides. —: not reported; GDM: gestational diabetes mellitus. Values are mean in mg/dL.

**Table 2:** Effect of hypercholesterolemia on tetrahydrobiopterin availability and endothelial function.

Study model	Tissue or cell type	Experimental condition	BH_4_ level	Parameter	Effect	Reference
Hypercholesterolemia	Human brachial artery	Basal	Reduced	Endothelium dependent vasodilation	Reduced	[13]
		BH_4_ infusion	Increased	Endothelium dependent vasodilation	Increased	

Hypercholesterolemia	Human coronary artery	Basal	Reduced	Coronary artery diameter and flow	Reduced	[51]
		BH_4_ infusion	Increased	Coronary artery diameter and flow	Increased	

Hypercholesterolemia	Human brachial artery	Basal	Reduced	Endothelium dependent vasodilation	Reduced	[52]
		BH_4_ supplementation	Increased	Endothelium dependent vasodilation	Increased	

Hypercholesterolemia	Human coronary microcirculation	Basal	nr	Myocardial blood flow	Reduced	[53]
		BH_4_ infusion	nr	Myocardial blood flow	Increased	

Hypercholesterolemia	Human skin	Basal	nr	Endothelium dependent vasodilation	Reduced	[54]
		R-BH_4_ infusion	nr	Endothelium dependent vasodilation	Increased	
		S-BH_4_ infusion	nr	Endothelium dependent vasodilation	Reduced	

Hypercholesterolemia	Human skin	Basal	nr	Endothelium dependent vasodilation	Reduced	[55]
		BH_4_ infusion	nr	Endothelium dependent vasodilation	Increased	

Cell culture	Human mesenteric microvascular endothelial cells	Incubation with oxLDL	Reduced	NO generation	Reduced	[56]
			Reduced	Superoxide generation	Increased	
		Incubation with oxLDL + sepiapterin	Increased	NO generation	Increased	
			Increased	Superoxide generation	Reduced	

Cell culture	Human aortic endothelial cells	Incubation with LDL	Reduced	NO generation	Reduced	[52]
		Incubation with LDL + BH_4_	nr	NO generation	Increased	

Basal corresponds to no treatment. BH_4_: tetrahydrobiopterin; R-BH_4_: R-tetrahydrobiopterin (NO synthase cofactor and antioxidant); S-BH_4_: stereoisomer of BH_4_ (antioxidant); oxLDL: oxidized low-density lipoprotein; LDL: low-density lipoprotein; NO: nitric oxide; nr: not reported.
